# COVID-19 Vaccination Coverage — World Health Organization African Region, 2021–2023

**DOI:** 10.15585/mmwr.mm7314a3

**Published:** 2024-04-11

**Authors:** Reena H. Doshi, Sheillah Nsasiirwe, Melissa Dahlke, Ajiri Atagbaza, Oniovo Efe Aluta, Alain Blaise Tatsinkou, Ezekiel Dauda, Alba Vilajeliu, Santosh Gurung, Jayne Tusiime, Fiona Braka, Ado Bwaka, Sarah Wanyoike, Donald J. Brooks, Diana Chang Blanc, James P. Alexander, Benjamin A. Dahl, Ann Lindstrand, Charles S. Wiysonge

**Affiliations:** ^1^Regional Office for Africa, World Health Organization, Brazzaville, Republic of the Congo; ^2^Global Immunization Division, Global Health Center, CDC; ^3^Department of Immunization, Vaccines and Biologicals, World Health Organization, Geneva, Switzerland.

**Summary**

**What is already known about this topic?**

The World Health Organization African Region did not receive enough COVID-19 vaccine doses to vaccinate everyone for whom vaccination was recommended and lagged behind other regions.

**What is added by this report?**

During 2021–2023, the cumulative number of COVID-19 vaccine doses received in the African Region increased from 321 million to 860 million, and 646 million doses were administered. Cumulative total population coverage with ≥1 dose ranged by country from 0.3% to 89%. By the end of 2023, coverage with a primary COVID-19 vaccination series increased from 7% to 32% for the total population, and increased to 52% among older age groups and to 48% among health care workers in a subset of countries in the African Region.

**What are the implications for public health practice?**

Additional outreach is needed to increase COVID-19 vaccination coverage among priority high-risk populations. Integrating COVID-19 vaccination into routine immunization and primary health care services could strengthen adult vaccination platforms and improve pandemic preparedness.

## Abstract

With the availability of authorized COVID-19 vaccines in early 2021, vaccination became an effective tool to reduce COVID-19–associated morbidity and mortality. Initially, the World Health Organization (WHO) set an ambitious target to vaccinate 70% of the global population by mid-2022. However, in July 2022, WHO recommended that all countries, including those in the African Region, prioritize COVID-19 vaccination of high-risk groups, including older adults and health care workers, to have the greatest impact on morbidity and mortality. As of December 31, 2023, approximately 860 million doses of COVID-19 vaccine had been delivered to countries in the African Region, and 646 million doses had been administered. Cumulatively, 38% of the African Region’s population had received ≥1 dose, 32% had completed a primary series, and 21% had received ≥1 booster dose. Cumulative total population coverage with ≥1 dose ranged by country from 0.3% to 89%. Coverage with the primary series among older age groups was 52% (range among countries = 15%–96%); primary series coverage among health care workers was 48% (range = 13%–99%). Although the COVID-19 public health emergency of international concern was declared over in May 2023, current WHO recommendations reinforce the need to vaccinate priority populations at highest risk for severe COVID-19 disease and death and build more sustainable programs by integrating COVID-19 vaccination into primary health care, strengthening immunization across the life course, and improving pandemic preparedness.

## Introduction

With the authorization and availability of highly effective COVID-19 vaccines by early 2021, vaccination became an effective tool to reduce COVID-19–associated morbidity and mortality worldwide. During 2021–2023, the COVID-19 Vaccines Global Access (COVAX), a global, multilateral initiative led jointly by Gavi, the Vaccine Alliance, the Coalition for Epidemic Preparedness Innovations, and the World Health Organization (WHO) in partnership with UNICEF, was established to ensure COVID-19 vaccine equity (*1*). To support the COVAX mission, increase population immunity, protect health systems, and facilitate economic recovery from the pandemic, WHO announced ambitious targets to administer a primary COVID-19 vaccination series to 10% of the total global population by the end of 2021 and 70% by mid-2022 (*2*). However, disparities in access to COVID-19 vaccines for low-income countries existed worldwide until the end of 2021, and a supply sufficient for effective rollout in the WHO African Region was therefore delayed until early 2022 (*3*). In July 2022, WHO recommended that all countries redirect efforts and focus on vaccinating priority populations, including health care workers, older adults (persons aged ≥50 years), and other high-risk groups (e.g., pregnant women, persons with comorbidities, and those with immunocompromising conditions) (*4*). This report provides an update on the progress made in COVID-19 vaccination in the African Region during 2021–2023.

## Methods

### Data Sources

The WHO African Region includes 47 of the 54 countries on the African continent^†^ with a total population of 1.2 billion based on individual country estimates (2023). Countries were requested to report weekly on the number of COVID-19 vaccine doses received from all sources and the number of doses administered. These data were compiled in the African Region regional database. Data from the regional database on COVID-19 vaccination during March 17, 2021–December 31, 2023, were used to assess COVID-19 vaccine supply and vaccination coverage among the total population^§^ and among high-priority groups. 

### Data Analysis

Vaccination coverage by country was calculated by dividing the number of persons who received a single dose or a complete primary series by the total population. Regional vaccination coverage was calculated by dividing the total number of persons who received a single dose or completed the primary series by the sum of the total population of every country in the region. Completion of primary series was defined as receipt of 1 or 2 doses depending on the vaccine product.^¶^ Booster doses were defined as any additional dose received among those who had completed the primary vaccination series, and booster dose coverage was calculated by dividing the number of persons who received ≥1 booster dose by the population who had completed a primary series. High-priority groups, as outlined by the WHO Strategic Advisory Group of Experts (SAGE) Roadmap for prioritizing use of COVID-19 vaccines, are groups for whom vaccines are of highest importance to reduce severe disease and death (*5*). The denominators for the total number of health care workers and older persons were reported individually by each country. This activity was reviewed by CDC, deemed not research, and was conducted consistent with applicable federal law and CDC policy.**

## Results

### Vaccine Supply

Because of global supply constraints in 2021, initial COVID-19 vaccine supply in the African Region was low, and vaccination was delayed. Ghana and Côte d’Ivoire were the first countries to receive small shipments of COVID-19 vaccine from COVAX in February and March 2021 (*6*). Availability began to improve in July; 321 million doses were received in the African Region during 2021, 449 million in 2022, and 90 million during 2023, for a cumulative total of 860 million doses (Table 1). Sixty-four percent of doses were acquired through the COVAX Facility, 15% by the African Union’s African Vaccine Acquisition Trust, 18% through bilateral agreements, and 2% through direct purchase from the manufacturer.

**TABLE 1:** COVID-19 vaccine doses received and primary series vaccination coverage — 47 World Health Organization African Region countries, 2021–2023

Year	Total no. of doses received (millions)	% Total vaccination coverage* (n = 46 countries)	% Vaccination coverage among older age groups^†^ (n = 22 countries)	% Vaccination coverage among health care workers
2021	**321**	7	NA	NA
2022	**449**	26	38	42^§^
2023	**90**	32	52	48^¶^

**Abbreviation:** NA = not available.

* The total population for each country was used as the denominator for vaccination coverage calculations. However, the eligible population for COVID-19 vaccination differed among countries; most countries targeted persons aged ≥16 or ≥18 years, but some countries vaccinated persons aged ≥5 years.

^†^ Older age group definition varies by country, but aged ≥50 years.

^§^ N = 29 countries.

^¶ ^N = 23 countries.

### Population Vaccination Coverage

By the end of 2023, 46 of the 47 countries^††^ in the African Region were delivering COVID-19 vaccination, and 646 million doses had been administered. Cumulatively, 440 million (37%) persons had received ≥1 dose (Figure). Cumulative primary series coverage increased from 7% in 2021 to 26% in 2022 and 32% in 2023. Cumulative total population coverage with ≥1 dose ranged by country from 0.3% to 89%. Coverage with ≥1 COVID-19 vaccine dose exceeded 70% in eight countries (Botswana, Cabo Verde, Liberia, Mauritius, Mozambique, Rwanda, Seychelles, and Sierra Leone; range = 72%–89%) (Table 2). Among these, Liberia, Mauritius, Rwanda, and Seychelles also achieved primary series coverage of ≥70% (range = 78%–86%). Conversely, 29 (62%) countries reported primary series coverage for the total population of <40%. Among the 40 countries reporting data on COVID-19 booster dose vaccination, coverage was 21%, varying widely among countries. Nine countries (Chad, Eswatini, Ghana, Mauritius, Namibia, Rwanda, Senegal, Seychelles, and Zimbabwe) achieved COVID-19 booster dose coverage of ≥40% of their total populations (range = 41%–81%).

**FIGURE:**
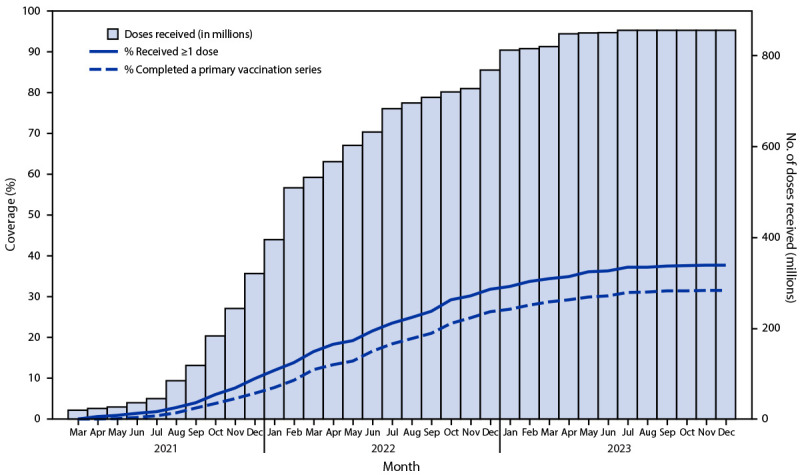
Cumulative number of COVID-19 vaccine doses received and cumulative ≥1-dose and primary series coverage among the total population,* by month — World Health Organization African Region, 2021–2023 * The total population for each country was used as the denominator for vaccination coverage calculations. However, the eligible population for COVID-19 vaccination differed among countries; most countries targeted persons aged ≥16 or ≥18 years, but some countries vaccinated persons aged ≥5 years.

**TABLE 2:** Cumulative COVID-19 vaccination coverage, by total population* and high-priority groups^†^ — World Health Organization African Region, March 2021–December 2023

Subregion	Country	At least 1 dose,* %	Primary series,* %	Booster dose, %	Health care workers, %	Older age groups,^§^ %
West Africa	Benin	28.7	21.3	NR	86.5	34.7
	Burkina Faso	27.4	23.6	0.1	86.3	NR
	Cabo Verde	72.6	62.8	0.2	NR	82.9
	Côte d’Ivoire	49.4	44.3	25.8	64.5	NR
	Ghana	43.7	34.0	44.6	80.5	41.7
	Guinea	65.7	44.1	7.3	NR	38.2
	Guinea-Bissau	36.5	26.7	13.9	90.7	NR
	Liberia	83.9	80.2	0.1	97.0	44.7
	Mali	20.6	17.2	NR	NR	50.5
	Mauritania	48.1	35.2	24.9	NR	NR
	Niger	25.5	22.4	NR	57.9	19.2
	Nigeria	43.3	37.5	21.1	32.9	NR
	Senegal	15.1	8.7	46.8	NR	39.5
	Sierra Leone	75.3	65.8	24.2	86.4	61.0
	The Gambia	25.5	20.4	10.0	NR	15.2
	Togo	28.3	19.5	27.5	88.1	NR
Central Africa	Angola	50.0	29.0	31.9	NR	NR
	Cameroon	13.5	11.5	25.1	NR	29.9
	Central African Republic	45.5	43.5	14.7	NR	24.2
	Chad	28.7	28.0	80.5	NR	41.1
	Democratic Republic of the Congo	14.3	12.1	NR	48.2	61.6
	Equatorial Guinea	16.5	13.1	2.1	70.5	NR
	Gabon	14.0	11.6	1.0	45.4	NR
	Republic of the Congo	12.0	11.3	NR	NR	NR
	Sao Tome and Principe	64.0	51.1	30.9	NR	NR
East and southern Africa	Algeria	17.7	14.7	8.9	NR	NR
	Botswana	79.2	67.5	30.7	NR	NR
	Burundi	0.3	0.3	0.9	12.9	NR
	Comoros	53.5	48.4	NR	72.9	NR
	Eritrea	NR	NR	NR	NR	NR
	Eswatini	45.3	36.8	42.0	NR	NR
	Ethiopia	49.0	40.7	13.7	57.0	96.3
	Kenya	27.9	21.4	18.0	53.3	51.0
	Lesotho	48.8	45.2	19.0	NR	NR
	Madagascar	9.3	9.1	6.4	56.2	15.8
	Malawi	28.1	22.2	29.2	NR	47.8
	Mauritius	88.8	86.0	60.1	NR	NR
	Mozambique	72.3	67.5	10.9	73.3	NR
	Namibia	24.7	21.6	54.2	58.4	29.6
	Rwanda	81.4	77.8	40.6	99.4	NR
	Seychelles	83.1	78.5	53.8	NR	NR
	South Africa	40.8	35.4	21.0	NR	67.0
	South Sudan	31.6	31.2	12.0	NR	NR
	Tanzania	56.2	52.5	NR	68.8	61.0
	Uganda	45.3	29.5	5.9	NR	37.8
	Zambia	61.8	48.3	15.5	NR	NR
	Zimbabwe	46.0	34.6	40.6	NR	NR

**Abbreviations: **NR = not reported; SAGE = Strategic Advisory Group of Experts on Immunization; WHO = World Health Organization.

* The total population for each country was used as the denominator for vaccination coverage calculations. However, the eligible population for COVID-19 vaccination differed among countries; most countries targeted persons aged ≥16 or ≥18 years, but some countries vaccinated persons aged ≥5 years.

^†^ High-priority groups as defined by the WHO SAGE roadmap for COVID-19 vaccination; data were not available for all specified groups.

^§ ^Older age group definition varies from country to country, but aged ≥50 years.

### Priority Population Vaccination Coverage

By the end of 2023, among the 23 countries reporting vaccination by high-risk population group, 48% of health care workers had completed the primary vaccination series; coverage with the primary series was ≥70% (range = 71%–99%) in 11 countries (Benin, Botswana, Comoros, Ghana, Guinea, Guinea-Bissau, Liberia, Mozambique, Rwanda, Sierra Leone, and Togo). Among 22 countries reporting data on older populations, primary series coverage was 52%. Only Cabo Verde and Ethiopia achieved coverage of ≥70% in this group. Coverage estimates among pregnant women and persons with comorbidities, including those with immunocompromising conditions, were unavailable because of incomplete reporting on these population categories.

## Discussion

Despite the improved supply of COVID-19 vaccine starting by late 2021, coverage in the African Region increased slowly. Regional coverage with a primary series reached 32% in 2023, with 38% of the population receiving ≥1 dose. Among the subset of countries that reported coverage for high-risk groups, 48% of health care workers and 52% of older adults received a primary series. Variation in coverage among countries was substantial. Four (9%) of the 47 countries in the region achieved the WHO target of 70% primary series coverage in the total population in 2022 (Liberia, Mauritius, Rwanda, and Seychelles); 29 (62%) countries reported primary series total population coverage <40%. Eritrea has not introduced COVID-19 vaccines, and Burundi delayed introduction in the general population and focused on vaccination of health care workers.

Several reasons likely account for low coverage with COVID-19 vaccines, including limited political commitment, logistical challenges, low perceived risk of COVID-19 illness, and variation in vaccine confidence and demand (*3*). Country immunization program capacity varies widely across the African Region. Challenges include weak public health infrastructure, limited number of trained personnel, and lack of sustainable funding to implement vaccination programs, exacerbated by competing priorities, including other disease outbreaks and endemic diseases as well as economic and political instability. The total population for each country was used as the denominator for vaccination coverage calculations. However, the eligible population for COVID-19 vaccination differed among countries; most countries targeted persons aged ≥16 or ≥18 years, but some countries vaccinated persons aged ≥5 years. In countries with large populations aged <18 years, meeting coverage targets was not possible (*7*).

Vaccination of high-priority groups remains critical for optimizing the impact of COVID-19 vaccines (*4*). Morbidity and mortality are highest among older adults and those with comorbidities (*5*), yet only two countries in the African Region have achieved >70% coverage among older age groups. The low coverage emphasizes the importance of targeted approaches to generate demand and address population concerns and of new delivery strategies to reach high-priority groups.

In May 2023, the public health emergency of international concern was officially declared over by WHO (*8*). In October 2023, SAGE recommended using a simplified primary vaccination series of a single dose of any COVID-19 vaccine and updated recommendations on revaccination for high-priority groups (*5*). SAGE recommended the continued prioritization of high-risk groups as described in the updated SAGE roadmap *(**5**)*. The recommendations also reinforced the need for sustainable programs and COVID-19 vaccination integration into primary health care and other relevant services. The aim was to optimize resources and build sustainable immunization delivery platforms throughout the life course in alignment with the Immunization Agenda 2030 goals (*9*).

In November 2023, the Regional Immunization Technical Advisory Group for the African Region endorsed the SAGE recommendations, encouraging countries to continue COVID-19 vaccination as aligned with national priorities (*10*). Many countries in the African Region are integrating COVID-19 vaccination into their routine health services and exploring new entry points for vaccinating high-priority populations as part of primary care and other relevant services, including through multiantigen periodic intensified routine immunization activities.

### Limitations

The findings in this report are subject to at least three limitations. First, immunization coverage estimates are based primarily on administrative data, which might contain inaccuracies resulting from errors in recording doses administered or in population estimates. Second, although reporting is highly encouraged, in 2023, many countries stopped reporting COVID-19 vaccination data because of competing priorities. In addition, fewer than one half of the countries are reporting doses administered among high-priority groups, including doses for health care workers and older persons. Finally, population estimates for high-priority groups are available only in some countries in the African Region, making assessing coverage challenging.

### Implications for Public Health Practice

The African Region has low COVID-19 vaccination coverage. Community engagement is needed to better understand drivers of vaccine confidence and develop more targeted strategies to improve vaccine demand (*4*). Integration of COVID-19 vaccination into routine immunization and primary health care services would help build sustainability and support recovery of routine immunization services (*9*). Strengthening adult immunization platforms would contribute to pandemic preparedness and global disease prevention goals (*4*). To protect vulnerable populations and prevent additional COVID-19 morbidity and mortality in the African Region, progress must continue to be made in vaccination of priority populations at highest risk for disease.

## Footnotes

* These authors contributed equally to this report.

† The WHO African Region includes the following countries: Algeria, Angola, Benin, Botswana, Burkina Faso, Burundi, Cabo Verde, Cameroon, Central African Republic, Chad, Comoros, Côte d’Ivoire, Democratic Republic of the Congo, Equatorial Guinea, Eritrea, Eswatini, Ethiopia, Gabon, Ghana, Guinea, Guinea-Bissau, Kenya, Lesotho, Liberia, Madagascar, Malawi, Mali, Mauritania, Mauritius, Mozambique, Namibia, Niger, Nigeria, Republic of the Congo, Rwanda, Sao Tome and Principe, Senegal, Seychelles, Sierra Leone, South Africa, South Sudan, Tanzania, The Gambia, Togo, Uganda, Zambia, and Zimbabwe.

§ The total population for each country was used as the denominator for vaccination coverage calculations. However, the eligible population for COVID-19 vaccination (the numerator for vaccination coverage calculations) differed among countries; most countries targeted persons aged ≥16 or ≥18 years, but some countries vaccinated persons aged ≥5 years.

¶ Primary series is defined as 1 dose of the Janssen (Jcovden) or Sputnik V/Light vaccines or 2 doses of AstraZeneca/Oxford or Serum Institute of India formulations (Vaxzevria and Covishield), Bharat Biotech (Covaxin), Moderna (Spikevax), Pfizer-BioNTech (Comirnaty), Sinopharm (Covilo), or Sinovac (CoronaVac) vaccines.

** 45 C.F.R. part 46, 21 C.F.R. part 56; 42 U.S.C. Sect.241(d); 5 U.S.C. Sect. 552a; 44 U.S.C. Sect. 3501 et seq.

†† Eritrea did not provide COVID-19 vaccines during 2021–2023.
